# In-ICU Outcomes of Critically Ill Patients in a Reference Cameroonian Intensive Care Unit: A Retrospective Cohort Study

**DOI:** 10.1155/2023/6074700

**Published:** 2023-05-08

**Authors:** Edgar Mandeng Ma Linwa, Charles Binam Bikoi, Joel Tochie Noutakdie, Emmanuel Ndoye Ndo, Jean Moise Bikoy, Charlotte Eposse Ekoube, Raissa Fogue Mogoung, Igor Simo Ghomsi, Michael Ngenge Budzi, Esther Eleonore Ngo Linwa, Martin Geh Meh, David Mekolo

**Affiliations:** ^1^Faculty of Health Sciences, University of Buea, Buea, Cameroon; ^2^Intensive Care Unit, Laquintinie Hospital Douala, Douala, Cameroon; ^3^Emergency Unit, Laquintinie Hospital Douala, Douala, Cameroon; ^4^Anaesthesiology Unit, Laquintinie Hospital Douala, Douala, Cameroon; ^5^Pediatric Unit, Laquintinie Hospital Douala, Douala, Cameroon; ^6^Faculty of Health Sciences, University of Bamenda, Bamenda, Cameroon

## Abstract

**Introduction:**

Mortality rate amongst critically ill patients admitted to the intensive care unit (ICU) is disproportionately high in sub-Saharan African countries such as Cameroon. Identifying factors associated with higher in-ICU mortality guides more aggressive resuscitative measures to curb mortality, but the dearth of data on predictors of in-ICU mortality precludes this action. We aimed to determine predictors of in-ICU mortality in a major referral ICU in Cameroon. *Methodology*. This was a retrospective cohort study of all patients admitted to the ICU of Douala Laquintinie Hospital from 1st of March 2021 to 28th February 2022. We performed a multivariable analysis of sociodemographic, vital signs on admission, and other clinical and laboratory variables of patients discharged alive and dead from the ICU to control for confounding factors. Significance level was set at *p* < 0.05.

**Results:**

Overall, the in-ICU mortality rate was 59.4% out of 662 ICU admissions. Factors independently associated with in-ICU mortality were deep coma (aOR = 0.48 (0.23–0.96), 95% CI, *p* = 0.043), and hypernatremia (>145 meq/L) (aOR = 0.39 (0.17–0.84) 95% CI, *p* = 0.022).

**Conclusion:**

The in-ICU mortality rate in this major referral Cameroonian ICU is high. Six in 10 patients admitted to the ICU die. Patients were more likely to die if admitted with deep coma and high sodium levels in the blood.

## 1. Introduction

Compared to high-income countries (HICs), optimal healthcare access is limited in low-and middle-income countries (LMICs) [1]. This is mainly due to poor health-seeking behaviors (preference of traditional medicine over conventional medicine), insufficient health facilities, lack of essential drugs, understaffed, and unfunded healthcare systems [2]. Consequently, late presentation of patients to the hospital are common and compounded with dreadful life-threatening complications of diseases which have a negative toll on in-hospital mortality [3]. The hospital department or ward best suited for managing these life-threatening diseases' complications often called critical illnesses is referred to as the intensive care unit (ICU) [4]. Sub-Saharan Africa (SSA) is one of the most poverty-stricken regions on earth, with an alarming double public health burden of communicable diseases (HIV/AIDS, malaria, tuberculosis, and hepatitis B and C infections) and an increasing incidence of noncommunicable diseases (hypertension, diabetes mellitus, and chronic kidney disease) stemming from the prevailing epidemiological transition of diseases. As a result, there is an increasing impact on the prevalence of critical illnesses seen in the ICU of SSA with a resultant disproportionately high in-ICU mortality in SSA [5].

Likewise, Cameroon, a SSA country dubbed “Africa miniature” is not spared by this high in-ICU death phenomenon compounded with limited ICU infrastructure and life-saving equipment [6]. For instance, the COVID-19 pandemic stormed Cameroon in 2020, when the national ICU capacity was 601 ICU beds and 73 mechanical ventilators for 23 million inhabitants [7]. The adverse effects of this low ICU capacity on in-ICU COVID-19 mortality cannot be overemphasized [8]. However, although in-ICU mortality rates are high in SSA [9], there is a dearth of data on the predictors of mortality especially in Cameroon. It is important to determine what factors may modify the fate of an individual admitted to the ICU in Cameroon. This will enable early implementation of appropriate preventive measures on modifiable parameters and closer monitoring in patients with nonmodifiable parameters. However, the fate of all cases, sometimes with similar pathology, is not the same [10]. This means some unidentified factors possibly contribute to mortality. Against this backdrop, we proposed this study to investigate in-ICU outcomes in a major referral Cameroonian ICU. As a preliminary study, this will generate reference baseline data on in-ICU mortality which researchers may explore in future related studies.

## 2. Methods

### 2.1. Study Design, Setting, and Participants

This was a retrospective cohort study carried out at the ICU of Douala Laquintinie Hospital (DLH) between March 1, 2021 and February 28, 2022. The period of follow-up was from admission at the ICU (t1) to discharge from the ICU or death (t2). We reviewed the medical records of all patients admitted to the ICU during the aforementioned study period. We excluded patients readmitted to the ICU during the same course of illness following an initial ICU discharge. We also excluded patients with incomplete data on ICU exit (discharged alive or dead) and patients aged <18years as this population usually has different mortality characteristics from the adult population. The DLH serves the population of the economic capital of Cameroon, Douala. The DLH is located in Douala I subdivision and receives averagely 150,000 patients per year. DLH serves as a referral centre where complex cases are sent sometimes at very late stages of diseases and serves a population with a high rate of poverty [11]. Care and approach to care is different from the other towns and other hospital facilities who receive more indolent cases at the initial stages of diseases and at a period when families and individuals are still financially viable. This is of utmost importance since Cameroon does not yet offer universal health coverage for its population, and healthcare is financed by individuals.

Its ICU is located next to the emergency and operating theatre. It was renovated in August 2021 from an 8-bed capacity unit to a 19-bed capacity unit. The unit is separated in to eight common rooms (two and three beds per room) and private rooms. Each room has a ventilator machine ready for utilisation in case of respiratory emergency requiring endotracheal intubation. Each bed has a cardiorespiratory monitor that measures blood pressure, displays an electrocardiogram, and oxygen saturation continuously. The unit provides level II basic ICU services which includes mechanical ventilation for longer than 24 h and specific organ support such as continuous vasopressor infusion. The staff is constituted of five anesthesiologists and intensive care physicians, three general practitioners, and 12 general nurses and nurse assistants. Every day, there are four nurses that take 12 hourly shifts, the general practitioners do rounds, and one anesthesiologist and intensive care physician ensures call coverage for the unit, the emergency department, and the anesthesiology unit. Two general practitioners, coming from other units of the hospital are available during each call at the ICU, based on a pre-established call program. One anesthesiologist is responsible for managing the emergency, general operating theatre, the maternity operating theatre, the ICU, and all other major emergencies in the different wards of the hospital. Based on individual needs, specialist consultants see patients routinely in association with the anesthesiologists and intensive care physicians. The hospital has a specialised centre where COVID-19 patients are managed and as such, patients with COVID-19 are never admitted to the general ICU ward described in the current study.

### 2.2. Study Sampling and Variables

Mortality event was the primary outcome selected for sample size calculation. The population included all eligible ICU patients and the outcome was dichotomous: alive or death. The exposure variable chosen was female sex as reported by Chukwuemeka O. Eze (27% among survivors and 46% among nonsurvivors) as a risk factor for the investigated outcome. The sample size was recalculated based on this information using the following information.

Where

 
*Z*_a_ represents standard normal variate for level of significance which is 1.96
 
*m* represents number of control subjects per experimental subject which is 1
 
*Z*_*b*_ represents standard normal variate for power or type 2 error which is 0.84
 
*p*1 represents probability of events in the control group which is 0.27
 
*p*2 represents probability of events in the experimental group which is 0.46
 
*p*^*∗*^=(*p*2+*mp*1)/(*m*+1) which represents the average proportion exposed

The minimum sample size required was 102 patients in each group. Thus, an overall minimum sample size of 204 patients was necessary for the study to have 80% power, significance of 5%, and 95% confidence interval [12].

A consecutive sampling method was used. Data were extracted from consultation registers, medical files, and laboratory reports into a predefined and pretested data collection form. These data included (1) sociodemographic data: age, sex, and marital status; (2) admission data: date of ICU admission, time of day of ICU admission, date of ICU discharge, and the hospital unit of origin of the patient; (3) clinical data: comorbidities, vital signs on ICU admission, clinical diagnoses, and the final ICU outcome (discharged alive or dead); and (4) laboratory data: white blood cell count, hemoglobin level, platelet count, sodium, potassium, urea, and creatinine in patient's blood samples. For this study, clinical diagnoses were categorised as sepsis without pneumonia, respiratory disorders, malignancy, pre/eclampsia/eclampsia/HELLP, severe head and neck injuries, coagulation disorders, metabolic disorders (hyperglycemic crisis and other endocrine disorders and electrolyte imbalance with dehydration), gastrointestinal bleeding disorders, cerebrovascular accidents, and others. For patients with multiple diagnoses, all clinical conditions were recorded as separate entities. As such, the number of diagnoses does not coincide with the number of patients as some patients had more than one diagnosis. Clinical scoring systems such as the SOFA (Sepsis-related Organ Failure Assessment) score were not used because of the unavailability of partial pressure of oxygen (PaO2), fraction of inspired oxygen (FiO2), and bilirubin levels in our sample. The quick SOFA score was an alternative, nonetheless, as this was a retrospective study, respiratory rates were seldom reported in the files studied.

### 2.3. Operational Definitions

Hypernatremia: sodium level in blood above 145 mEq/L
Hyperglycemia: glucose level in blood above 200 mg/dL
Sepsis without pneumonia: All cases of sepsis except cases of pneumonia
Respiratory disorders: All cases of respiratory illnesses including pneumonia, pulmonary oedema, pulmonary embolism, etc.
  (1) $$\begin{array}{c} Mortality\;rate≔\frac{number\;of\;cases\;admitted\;at\;the\;ICU\;that\;died}{total\;number\;of\;ICU\;admissions}\text{.} \end{array}$$
Deep coma: any patient with a Glasgow coma score level ≤8/15
Late Night deaths: It refers to the category of patients who died between midnight and 6:00 am.

### 2.4. Statistical Considerations and Data Analysis

Data were entered in Microsoft Excel 365 and analyzed using R version 4.1.1. Distribution of socio-demographic, admission, clinical, and laboratory data were studied and compared between patients discharged alive and dead from the ICU. Continuous data were checked for normality using the Shapiro-Wilk test and summarized as means with standard deviations (SDs) if data was normally distributed or as medians and interquartile ranges (IQRs) if data was not normally distributed. Frequencies and proportions were calculated for categorical data. Missing values and outliers were identified using explorative data analysis. The Chi-square test was used to compare categorical variables unless more than 20% of expected cell counts were less than 5, in which case, Fisher's exact test was employed. Multivariable analysis was used to determine the effects of independent variables on the outcome variable of ICU “discharged alive” dichotomized as a yes/no variable. Independent variable selection was informed by review of relevant scientific literature of predictors of ICU mortality in similar contexts and data availability. All independent variables with *p* values less than 0.05 were included in the multivariable models, built in a stepwise fashion, and multicollinearity was checked using the variance inflation factor (VIF), excluding any variable with a VIF greater than 10. Missing value categories were created in categorical variables before including in logistic regression model. Adjusted odds ratios (aOR), their corresponding 95% confidence intervals (95% CI), and *p* values were reported. The level of statistical significance was set at *p* < 0.05.

### 2.5. Ethical Considerations and Administrative Clearance

Administrative clearance was obtained from the Directorate of the DLH. For the following reasons, the authors decided not to request for informed consent or ethical committee approval for their study: All studied data were gathered as part of standard medical diagnosis and care. Patients were also diagnosed and treated in accordance with national standards and agreements. Since this was a retrospective study, informed consent was not necessary.

## 3. Results

### 3.1. Patients Flow Chart

**Figure 1:**
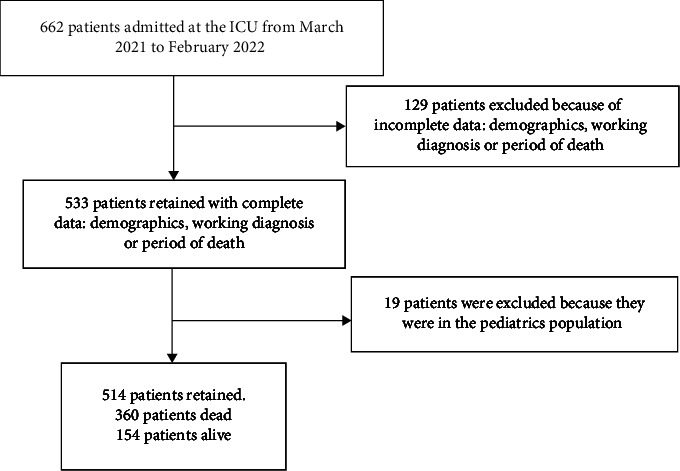
Flow chart of patient inclusion in the study.

Overall, the medical records of 662 critically ill patients were reviewed. A total of 129 records were excluded due to incomplete data (demographics, working diagnosis, or period of death). Subsequently, 19 patients were excluded because they were in the pediatrics population. Hence, 514 patients were retained as the study population as shown in Figure 1.

### 3.2. General Characteristics of the Study Population

The median age of the sample was 49 years (Q1–Q3: 34.2–64.0). Patient's age ranged from 18 years to 97 years. The young adults (18–40 years) constituted 34.8% (*n* = 179), older adults (41–60 years) constituted 33.3% (*n* = 171), and the elderly (>60 years) constituted 31.9% (*n* = 164) of the population. The median duration of ICU stay was 3.0 days (Q1–Q3: 2.0–5.0). The minimum duration of ICU hospitalisation was 1 day with a maximum of 94 days. There were 51.2% (*n* = 263/514) males, giving a male to female sex ratio of 1.05 : 1. A total of 49.2% of the sample was married. Patients were admitted most in the month of August 2021, that is, 11.8% (*n* = 78/662) of the sample. For comorbidities, they were reported in 48.5% of the sample. HIV was found in 8.9% (*n* = 46), diabetes in 16.9% (*n* = 87), malignancy in 4.3% (*n* = 22), and hypertension in 19.5% (*n* = 100). The unit of origin of half of the patients (54.1%) admitted to the ICU was the emergency department, 11.5% from surgical ward, 10.7% from the medical wards, 8.6% from maternity ward, 8.4% from the operating theatre, 4.3% from mixed medical-surgical wards, and 2.1% received directly from the ambulance referred from another hospital. The most common reason for transfer to the ICU was coma (83.9%), followed by respiratory distress, severe sepsis, hemodynamic instability, postoperative monitoring, convulsions/agitation, and pre-eclampsia/eclampsia (5.6%) as depicted in Table 1. The most common diagnosis was sepsis excluding pneumonia (38.6%), followed by respiratory disorders (27.4%), metabolic disorders (23.9%), cerebrovascular accidents (13.9%), severe head injury (13.6%), meningitis/meningoencephalitis (11.1%), coagulation disorders (5.8%), pre-eclampsia (5.3%), malignant disorders (5.3%), and gastrointestinal bleeding (4.7%) as seen in Table 1. Most patients (54.9%, *n* = 300) were admitted between 6 am and 6 pm.

### 3.3. Mortality Analysis

**Figure 2:**
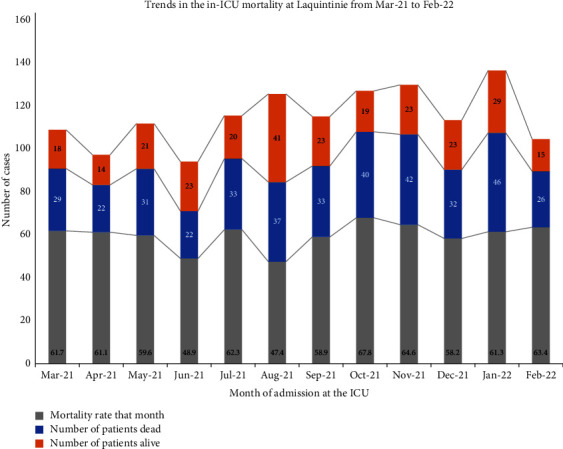
Trends in mortality at the ICU laquintinie.

The overall in-ICU mortality rate was 59.4% (*n* = 393/662). The month with the highest mortality was October 2021 (67.8%, *n* = 40/59). Conversely, the month with the lowest mortality was August 2021 with 47.4% (*n* = 37/78) of deaths as shown in Figure 2. The lowest number of admissions was in April 2021 (*n* = 36) and the highest number of admissions in August 2021 (*n* = 78). Overall, the proportion of deaths occurring at night (6 pm to 6 am) was 43.3% (156/360) with late night deaths (midnight to 6 am) accounting for 25% of overall death (92/360).

Univariate analysis identified sociodemographic and clinical characteristics such as mean age (*p* < 0.001), age >40 years (*p* < 0.001), male gender (*p*=0.047), emergency ward admissions (*p* < 0.001), maternity ward admissions (*p* < 0.001), coma (*p* < 0.001), postoperative monitoring (*p*=0.002), hemodynamic instability (*p*=0.032), median diastolic blood pressure (*p*=0.003), median temperature (*p*=0.014), median oxygen saturation (*p*=0.004), median Glasgow coma scale score (*p* < 0.001), deep coma (*p* < 0.001) pre-eclampsia/eclampsia/HELLP syndrome (*p* < 0.001), cerebrovascular accidents (*p*=0.014), coagulation disorders (*p*=0.017), and transfusions (*p*=0.043) to be significantly associated with mortality in the ICU. Some paraclinical characteristics also significantly associated with mortality in the ICU were sodium level (*p*=0.033), hypernatremia (*p*=0.024), urea level (*p*=0.042), and creatinine level (*p*=0.015) as shown in Table 2.

Multivariable analysis showed only two factors were significantly associated with mortality: deep coma (aOR = 0.48 (0.23–0.96), 95% CI, *p* = 0.043), and hypernatremia (>145 meq/L) (aOR = 0.39 (0.17–0.84) 95% CI, *p* = 0.022) on admission as shown in Table 3.

## 4. Discussion

This study aimed to determine the predictors of in-ICU mortality in a major referral ICU of Cameroon, with scarce previous data on this subject. Factors found to be independently associated with in-ICU mortality were deep coma and hypernatremia. The overall mortality rate in the current study was 59.4% (*n* = 393/662). This is much higher than that described previously in Cameroon (7.8–8.18%) [13, 14], Malawi (20.6%) [15], and Uganda (27%) [16, 17]. Nonetheless, studies carried out in Nigeria and Ethiopia [18, 19] reported mortality rates (40.8–61.4%) much similar to those which we obtained in our current study. The studies reported in Cameroon [13, 14] have a very low mortality rate because they were carried out in special ICU centers receiving only pediatric and obstetric and gynecological cases. Medical and visceral surgical pathologies are not admitted in that ICU, thus pathologies such as cerebrovascular accidents, severe sepsis from medical pathologies, burns, or even unstable postoperative patients with visceral pathologies are not admitted, constituting a great bias for the appraisal of the reported ICU mortality. Moreover, the low mortality reported in Uganda can be explained by the readily available respiratory support and by the fact that 87% of hospital patient costs are subsidized. Cost of care has been recognised as a great hinderance to optimal care in the ICU [20].

The top 5 deadliest pathologies in our unit were cerebrovascular accidents (81.9%) followed by coagulation disorders (78.8%), meningitis/meningoencephalitis (77.0%), respiratory disorders (73.1%), and severe sepsis excluding pneumonia (72.1%). In Malawi, it has been noted that the highest mortality was among patients admitted with sepsis (59.3%) [15]. In Nigeria, a similar pattern has been described with acute diabetic complications having the worst case fatality rate (100%), followed by cerebrovascular accidents (71%), renal failure (62.5%), cancer (60%), sepsis (55.5%), acute abdomen (34%), and trauma (23%) [18]. Similar to our study, cerebrovascular accidents have also been reported by Mbengono et al. [13] as the most important cause of mortality in the ICU in Cameroon. Metabolic disorders in our study which included acute diabetic conditions such as diabetic ketoacidosis, severe hypoglycemia, nonketotic hyperglycemic crisis as well as ionic imbalance, and hepatic encephalopathy had a much lower case fatality ratio (67.9%) than that reported by Eze et al. [18]. The least deadly pathology in our study was pre-eclampsia/eclampsia/HELLP syndrome with a case-fatality ratio (CFR) of 9.7% (*n* = 3/34) as similarly reported by Bhagwanjee et al. [21] from South Africa who obtained a CFR of 10.5%. This is much lower than reported by Priso et al. [22] from Cameroon (24.3%). This high mortality rate is probably because pre-eclampsia was not well known by the medical personnel and the population in the 2007–2014 era, when this study was carried out. Moreover, at that time, patients usually presented late. More awareness and strict national guidelines to reduce maternal mortality put in place by the Ministry of Health have probably contributed to limit this mortality as noted in our study.

In multivariable analysis, deep coma (GCS ≤ 8) and hypernatremia >145 meq/L) were associated with mortality. A study carried out in Brazil also reported electrolyte disturbance as being associated with increased mortality [23]. Even in more advanced settings such as Austria, hypernatremia has been reported as an independent risk factor for mortality (relative risk, 2.1) [24]. Hypernatremia can arise from a variety of causes, such as instances where attempts are made to rapidly correct hyponatremia in emergency settings, but most often than not is a marker of severity of underlying pathologies [25]. Patients with blood sodium levels above 150 mmol/L have a mortality rate between 30 and 48 percent, according to some studies carried out on the potential effects of hypernatremia on outcomes in critically sick patients in a medical ICU [26–28]. Relating to coma, a study carried out by Bekele et al. in Ethiopia and Mbengono et al. in Cameroon, reported that patients admitted with a Glasgow coma scale <9 were more likely to die [29, 30]. This is understandable since a low GCS reflects the severity of neurological impairment and reduced patient autonomy, making the patient more dependable and requiring closer monitoring [31].

Minkande et al. in Cameroon has reported 55% of deaths to occur between 8 p.m. and 8 a.m and postulated that this probably relates to poor compliance with treatment, staff shortages, and insufficient monitoring equipment [14]. Contrary to these results, the proportion of deaths occurring at night (6 pm to 6 am) was 43.3% (156/360) with late night deaths (midnight to 6 am) accounting for 25% of overall death (92/360). Though staffing at our ICU was equally reported to be lower at night, proportions of deaths during the day and the night were similar suggesting a minimal effect of such variables on the overall mortality.

Although scoring systems were not used in our study, several tools could have been useful for risk assessment such as the APACHE score [32–34] and the Simplified Acute Physiology Score 3 (SAPS 3) which have already been tested in African settings [35, 36] despite the major limitation of relying on paraclinical parameters seldom available in sub-saharan Africa. Scoring systems that are mostly or entirely clinical such as the Modified Early Warning Score (MEWS) [37] are more adapted to developing countries, although some authors have reported that this particular score is poor at predicting ICU outcomes [38]. Other scores used in patients with sepsis include the Rwanda Mortality Probability Model (R-MPM), the Universal Vital Assessment (UVA) score, and the qSOFA score, which have similar discriminating capacities in low-resource settings [39]. These scores were not used in the current study since we were evaluating all-cause mortality and not only sepsis-related mortality for which these scores were created and validated. However, these scores can be used in further projects.

This study highlights findings for actionable recommendations. We recommend to practitioners to be prompt in requesting sodium level and keen in managing electrolyte imbalances prior to ICU referral. Moreover, practitioners should be prompt in referring patients with altered consciousness and not wait for the Glasgow coma score to be below 9 before referral. Further studies are encouraged to give us more insight in to these assumptions.

A very significant limitation of the current study is that factors that could have significantly contributed in the evaluation of disease severity such as the use of life-sustaining therapies including oxygen (nasal prongs or face mask), noninvasive ventilation, CPAP, high-flow nasal oxygen, and invasive organ support such as mechanical ventilation, blood gas measurements, renal replacement therapy, or vasopressor therapy could not be retrieved because of the retrospective nature of the current study. Still owing to the retrospective nature of this study, a significant proportion (19.5%) of patients were excluded because of missing data. This could have contributed in creating selection bias in our study and authors strongly suggest that a prospective design should be employed in future studies to confirm or refute our findings. Moreover, this study reports survival to ICU discharge but not survival to hospital discharge. The former mortality rates may not represent the real mortality rate as survival to ICU discharge is usually very different from survival to hospital discharge, which is a better assessment gauge of treatment efficacy [40, 41]. Additionally, no analyses were performed to assess for possible unmeasured confounding. Furthermore, this study cannot be generalised because it was carried out at a single centre in an urban setting with peculiar populations.

Nonetheless, this is one of the largest scale studies (*n* > 500 patients) evaluating in-ICU mortality in SSA and taking into consideration important, but often overlooked crucial parameters such as laboratory data (hemoglobin level, serum creatinine, and serum electrolytes) generally unaffordable or relatively expensive in this resource-limited area. The study was carried out in a secondary level facility with the greatest number of admissions in Cameroon and represented real-life evaluation on the need for ICU services and provides a guide to reduce mortality and increase the efficiency of these services.

## 5. Conclusion

ICU mortality rates in developing countries are high and Cameroon is not an exception. Six in 10 patients admitted to the ICU died. Patients were more likely to die if admitted with deep coma and high serum sodium levels. Further studies need to be carried out to enquire about nondisease-specific factors that could contribute to mortality such as staff competence and workload, availability of care, ICU equipment, health insurance status and financial viability.

### 5.1. What Is Known on This Topic?

- In sub-Saharan Africa (SSA), patients tend to present late to the hospital with dreadful life-threatening disease complications which require ICU admissions
- There is an increasing impact on the prevalence of critical illnesses seen in the ICU with a resultant disproportionately high in-ICU mortality in SSA
- Although in-ICU mortality rates are high in sub-Saharan Africa, there is a dearth of data on the predictors of mortality especially in Cameroon. It is important to determine what factors may modify the fate of an individual admitted to the ICU in Cameroon.

### 5.2. What Does This Study Add?

- ICU mortality rates in developing countries are high with six in 10 patients admitted that die.
- Patients were more likely to die if admitted with deep coma (GCS ≤8) and high serum sodium levels (>145 mEq/L).
- This risk group was represented as such: 30.2% of patients admitted with deep coma (GCS ≤8) and 20.8% of patients admitted with hypernatremia >145 mEq/L

## Figures and Tables

**Table 1:** Baseline sociodemographic and clinical characteristics of patients admitted at ICU laquintinie.

Variables	No death *n* = 154 (%)	Death *n* = 360 (%)	Total *n* = 514	*p* value
Median age in years (SD)	39.0 (27.0–56.8)	53.0 (39.0–65.9)	49.0 (34.2–64.0)	**<0.001**
*Age category*				
>40 years	74 (48.1)	261 (72.5)	335 (65.2)	**<0.001**
Marital status (married)	73 (47.4)	180 (50.0)	253 (49.2)	0.129
Male gender	68 (44.2)	195 (54.2)	263 (51.2)	**0.047**
*Medical history*				
Cancer	5 (3.2)	17 (4.7)	22 (4.3)	0.604
HIV	8 (5.2)	38 (10.6)	46 (8.9)	0.075
Diabetes	24 (15.6)	63 (17.5)	87 (16.9)	0.688
Hypertension	22 (14.3)	78 (21.7)	100 (19.5)	0.070
*Origin of patients*				
Emergency ward	69 (44.8)	209 (58.1)	278 (54.1)	<0.001
Surgical ward	20 (13.0)	39 (10.8)	59 (11.5)	0.242
Internal medicine ward	12 (7.8)	43 (11.9)	55 (10.7)	0.081
Maternity ward	31 (20.1)	13 (3.6)	44 (8.6)	**<0.001**
Operating theatre	12 (7.8)	31 (8.6)	43 (8.4)	0.388
Mixed wards	4 (2.6)	18 (5.0)	22 (4.3)	0.112
Direct entry	4 (2.6)	7 (1.9)	11 (2.1)	0.318
*Reasons for transfer*				
Coma	97 (63.0)	318 (88.3)	431 (83.9)	**<0.001**
Respiratory distress	79 (51.3)	189 (52.5)	268 (52.1)	0.878
Severe sepsis	58 (37.7)	161 (44.7)	219 (42.6)	0.112
Hemodynamic instability	18 (11.7)	76 (21.1)	94 (18.3)	**0.012**
Postoperative monitoring	40 (26.0)	53 (14.7)	93 (18.1)	**0.003**
Convulsions/agitations	29 (18.8)	53 (14.7)	82 (16.0)	0.307
*Diagnosis*				
Severe sepsis excluding pneumonia	52 (33.8)	145 (40.7)	197 (38.6)	0.113
Respiratory disorders	36 (23.4)	105 (29.2)	141 (27.4)	0.190
Metabolic disorders	37 (24.0)	86 (23.9)	123 (23.9)	0.484
Cerebrovascular accident	12 (7.8)	59 (16.6)	71 (13.9)	**0.014**
Severe head injury	20 (13.0)	50 (13.9)	70 (13.6)	0.398
Meningitis/meningoencephalitis	11 (7.1)	46 (12.8)	57 (11.1)	0.061
Coagulation disorders	4 (2.6)	26 (7.2)	30 (5.8)	**0.017**
Pre-eclampsia/eclampsia	24 (15.6)	3 (0.8)	27 (5.3)	**<0.001**
Malignant disorders	5 (3.2)	22 (6.1)	27 (5.3)	0.092
Gastrointestinal bleeding	7 (4.5)	17 (4.7)	24 (4.7)	0.235
*Outcomes*				
Transfusion received	48 (31.2)	80 (22.2)	128 (24.9)	**0.043**
Hospitalisation >3 days	105 (68.2)	112 (31.1)	217 (42.2)	**<0.001**
Median duration of hospitalisation in days (SD)	5.0 (3.0–7.0)	2.0 (1.0–4.0)	3.0 (2.0–5.0)	**<0.001**

Bold values represent variables with statistical significance *p* < 0.05.

**Table 2:** Clinical and paraclinical parameters on admission (univariate analysis).

Continuous variables	No death *n* = 154 median (Q1–Q3)	Death *n* = 360 median (Q1–Q3)	Total *n* = 514 median (Q1–Q3)	*p* value

Vital signs				
Systolic BP (mmHg)	135.0 (115.0–156.0)	129.0 (103.8–156.2)	131.0 (107.2–156.0)	0.064
Diastolic BP (mmHg)	83.0 (70.0–98.0)	76.0 (62.0–93.0)	78.0 (65.0–93.8)	**0.003**
Glucose levels (mg/dL)	132.5 (108.0–169.0)	139.0 (110.0–195.0)	137.0 (110.0–189.0)	0.268
Temperature (C)	37.5 (37.0–38.4)	38.0 (37.0–39.0)	37.8 (37.0–38.8)	**0.010**
Oxygen saturation (%)	95.0 (90.0–97.0)	93.0 (87.0–97.0)	94.0 (89.0–97.0)	**0.004**
Pulse (beats per minute)	105.0 (89.0–120.8)	110.0 (87.0–126.0)	107.5 (88.0–124.0)	0.607
Glasgow coma scale score	12 (8.0–15.0)	9.0 (6.0–12.0)	10.0 (7.0–14.0)	**<0.001**
Paraclinical parameters				
White blood cell count	10.4 (7.5–14.3)	10.0 (6.6–14.6)	10.3 (7.0–14.6)	0.407
Haemoglobin level	10.6 (8.9–12.6)	10.5 (8.7–12.7)	10.5 (8.7–12.7)	0.904
Platelet count	191.5 (120.2–287.0)	189.5 (115.0–267.2)	190.0 (116.0–268.0)	0.650
Sodium (mEq/L)	138.1 (136.0–142.0)	139.9 (135.9–145.0)	139.4 (135.9–143.6)	**0.033**
Potassium (mEq/L)	3.9 (3.4–4.4)	3.8 (3.3–4.5)	3.8 (3.3–4.5)	0.754
Urea level (g/L)	0.4 (0.3–0.9)	0.6 (0.3–1.3)	0.5 (0.3–1.2)	**0.042**
Creatinine level (mg/dL)	1.28 (1.01–1.94)	1.6 (1.05–3.26)	1.45 (1.02–2.9)	**0.015**

Categorical variables	No death *n* = 154 counts (%)	Death *n* = 360 counts (%)	Total *n* = 514 counts (%)	*p* value

Vital signs				
Hypotension (SBP <90)	10 (6.5)	38 (10.6)	48 (9.3)	0.199
Fever >40°C	1 (0.6)	26 (7.2)	27 (5.3)	**0.004**
Tachycardia >100 bpm	89 (57.8)	208 (57.8)	297 (57.8)	1.000
Hypoxia (<90%)	27 (17.5)	104 (28.9)	131 (25.5)	**0.007**
Deep coma (GCS ≤8)	26 (16.9)	129 (35.8)	155 (30.2)	**<0.001**
Hypoglycemia <70 g/L	5 (3.2)	15 (4.2)	20 (3.9)	0.806
Hyperglycemia >200 g/L	26 (16.9)	83 (23.1)	109 (21.2)	0.147
Paraclinical parameters				
^a^WBC count ≥15 × 109/mL	32 (23.9)	63 (24.0)	95 (23.9)	1.000
^a^Severe anemia ≤7 g/L	13 (9.6)	28 (10.6)	41 (10.3)	0.897
^a^STP <50,000	3 (2.2)	18 (6.9)	21 (5.3)	0.087
^b^Hypernatremia >145 meq/L	12 (12.6)	47 (25.0)	59 (20.8)	**0.018**
^b^Hyperkaliemia >6 mEq/L	4 (4.2)	13 (6.9)	17 (6.0)	0.523
^c^Hyperuremia >2 g/L	6 (5.8)	26 (12.0)	32 (9.9)	0.105
^c^Hypercreatinemia >40 mg/L	11 (10.2)	41 (18.6)	52 (15.8)	0.073

Note: WBC = white blood cell, SBP = systolic blood pressure, STP = severe thrombocytopenia. a = 397 patients, b = 284 patients, c = 323 patients. Bold values represent variables with statistical significance *p* < 0.05.

**Table 3:** Multivariable analysis model comparing mortality risk in the sample.

Variables	OR (95% CI)	*p*-value	aOR (95% CI)	*p*-value
Sociodemographic characteristics				
Median age	0.97 (0.96–0.98)	**<0.001**	0.99 (0.97–1.01	0.299
Male gender^a^	0.67 (0.46–0.98)	**0.047**	1.70 (0.85–3.46)	0.137

Origin of patients				
Maternity ward^b^	7.22 (3.65–15.03)	**<0.001**	2.74 (0.48–17.26)	0.258

Reasons for transfer				
Postoperative monitoring	2.04 (1.28–3.24)	**0.003**	1.42 (0.64–3.15)	0.386
Hemodynamic instability	0.49 (0.28–0.84)	**0.012**	0.67 (0.28–1.51)	0.344

Diagnosis				
Cerebrovascular accident	0.43 (0.21–0.80)	**0.011**	0.62 (0.24–1.52)	0.314
Pre-eclampsia/eclampsia	21.72 (7.44–92.49)	**<0.001**	6.37 (0.75–139.15)	0.128

Clinical parameters^d^				
Median diastolic BP (mmHg)	1.01 (1.00–1.02)	**0.003**	1.01 (1.00–1.02)	0.123
Hypoxia	0.52 (0.32–0.83)	**0.007**	0.73 (0.37–1.40)	0.344
Median temperature	0.82 (0.70–0.95)	**0.010**	1.17 (0.91–1.49)	0.218
Deep coma (GCS ≤8)	0.36 (0.22–0.58)	**<0.001**	0.48 (0.23–0.96)	**0.043**
Hypernatremia	0.43 (0.21–0.84)	**0.018**	0.39 (0.17–0.84)	**0.022**

Note: OR = odds ratio. CI = confidence interval, aOR = Adjusted odds ratio. ^a^Reference category is female gender. ^b^Reference category is emergency ward admissions. Mean variance inflation factor (VIF): 1.26. Bold values represent variables with statistical significance *p* < 0.05.

## Data Availability

The data generated in this article are available from the corresponding author upon reasonable request.

## References

[1] Savigny D., Adam T. (2009). Research A for HP and S, Organization WH. Systems Thinking for Health Systems Strengthening. https://apps.who.int/iris/handle/10665/44204.

[2] Bright T., Felix L., Kuper H., Polack S. (2017). A systematic review of strategies to increase access to health services among children in low and middle income countries. *BMC Health Services Research*.

[3] Simbila A. N., Kilindimo S. S., Sawe H. R. (2022). Predictors and outcome of time to presentation among critically ill paediatric patients at Emergency Department of Muhimbili National Hospital, Dar es Salaam, Tanzania. *BMC Pediatrics*.

[4] Marshall J. C., Bosco L., Adhikari N. K. (2017). What is an intensive care unit? A report of the task force of the World Federation of Societies of Intensive and Critical Care Medicine. *Journal of Critical Care*.

[5] Gouda H. N., Charlson F., Sorsdahl K. (2019). Burden of non-communicable diseases in sub-saharan Africa, 1990–2017: results from the global burden of disease study 2017. *Lancet Global Health*.

[6] Papali A., Adhikari N. K. J., Diaz J. V., Dondorp A. M., Dünser M. W., Jacob S. T., Dondorp A. M., Dünser M. W., Schultz M. J., éditeurs (2019). Infrastructure and organization of adult intensive care units in resource-limited settings [internet]. *Sepsis Management in Resource-Limited Settings*.

[7] Metogo J. A. M., Tochie J. N., Etoundi P. O., Bengono R. S. B., Ndikontar R., Jemea B. (2020). The Intensive Care Capacity for Severe Forms of COVID-19 in Africa: Why Is Africa Not Making Progress Faced with This Pandemic? J. Xiangya Med. https://jxym.amegroups.com/article/view/6431.

[8] Mekolo D., Bokalli F. A., Chi F. M., Fonkou S. B., Takere M. M., Ekukole C. M. (2021). Clinical and Epidemiological Characteristics and Outcomes of Patients Hospitalized for COVID-19 in Douala, Cameroon. *Pan Afr. Med. J*.

[9] Abate S. M., Assen S., Yinges M., Basu B. (2021). Survival and predictors of mortality among patients admitted to the intensive care units in southern Ethiopia: a multi-center cohort study. *Annals of medicine and surgery (2012)*.

[10] Mayr V. D., Dünser M. W., Greil V. (2006). Causes of death and determinants of outcome in critically ill patients. *Critical Care*.

[11] Fambon S. (2006). *A Dynamic Poverty Profile For Cameroon*.

[12] Dean A. G., Sullivan K. M., Soe M. M. (2013). OpenEpi: Open Source Epidemiologic Statistics for Public Health. https://www.openepi.com/Menu/OE_Menu.htm.

[13] Mbengono J. A. M., Bengono R. B., Nkodo J. M., Essame T. C., Amengle A. L., Minkande J. Z. (2015). Etiologies des décès dans les services d’urgences et de réanimation dans deux hôpitaux de la ville de Yaoundé. *Health Sci. Dis*.

[14] Minkande J. Z., Chiabi A., Mboudou E., Obeme H., Tehem J., Bilounga E. (2007). Etude de la mortalite dans le service de eanimation de l\’hopital gyneco-obstetrique et pediatrique de Yaounde. Clin. Mother Child Health. https://www.ajol.info/index.php/cmch/article/view/35870.

[15] Prin M., Itaye T., Clark S. (2016). Critical care in a tertiary hospital in Malawi. *World Journal of Surgery*.

[16] Towey R., Ojara S. (2008). Practice of intensive care in rural Africa: an assessment of data from Northern Uganda. *African Health Sciences*.

[17] Towey R. M., Ojara S. (2007). Intensive care in the developing world. *Anaesthesia*.

[18] Eze C. O., Okoro F. C., Nnaji T., Nwobodo M., Kalu U., Ewah R. (2020). Mortality pattern in intensive care unit: experience at abakaliki southeastern Nigeria. *World Journal of Cardiovascular Diseases*.

[19] Smith Z. A., Ayele Y., McDonald P. (2013). Outcomes in critical care delivery at jimma university specialised hospital, Ethiopia. *Anaesthesia & Intensive Care*.

[20] van der Sluijs A. F., van Slobbe-Bijlsma E. R., Chick S. E., Vroom M. B., Dongelmans D. A., Vlaar A. P. J. (2017). The impact of changes in intensive care organization on patient outcome and cost-effectiveness—a narrative review. *J. Intensive Care*.

[21] Bhagwanjee S., Paruk F., Moodley J., Muckart D. J. J. (2000). Intensive care unit morbidity and mortality from eclampsia: an evaluation of the acute Physiology and chronic health evaluation II score and the Glasgow coma scale score. *Critical Care Medicine*.

[22] Priso E. B., Njamen T. N., Tchente C. N., Kana A. J., Landry T., Niaga F. (2015). Trend in Admissions, Clinical Features and Outcome of Preeclampsia and Eclampsia as Seen from the Intensive Care Unit of the Douala General Hospital, Cameroon. *Pan Afr. Med. J*.

[23] Soares Pinheiro F. G., Santana Santos E., Barreto Í. D. (2020). Mortality predictors and associated factors in patients in the intensive care unit: a cross-sectional study. *Critical Care Research and Practice*.

[24] Lindner G., Funk G. C., Schwarz C. (2007). Hypernatremia in the critically ill is an independent risk factor for mortality. *American Journal of Kidney Diseases*.

[25] Lindner G., Funk G. C. (2013). Hypernatremia in critically ill patients. *Journal of Critical Care*.

[26] Kraft M. D., Btaiche I. F., Sacks G. S., Kudsk K. A. (2005). Treatment of electrolyte disorders in adult patients in the intensive care unit. *American Journal of Health-System Pharmacy*.

[27] Darmon M., Timsit J. F., Francais A. (2010). Association between hypernatraemia acquired in the ICU and mortality: a cohort study. *Nephrology Dialysis Transplantation*.

[28] Hoorn E. J., Betjes M. G. H., Weigel J., Zietse R. (2008). Hypernatraemia in critically ill patients: too little water and too much salt. *Nephrology Dialysis Transplantation*.

[29] Bekele Z., Jisha H., Haile M. (2022). Outcomes and associated factors among adult patients admitted to adult intensive care unit, retrospective cohort study. *International Journal of Surgery: Oncology*.

[30] Mbengono J. A. M., Tochie J. N., Ntock F. N. (2019). The epidemiology, therapeutic patterns, outcome, and challenges in managing septic shock in a sub-saharan african intensive care unit: a cross-sectional study. *Hosp. Pract. Res.*.

[31] Cooksley T., Rose S., Holland M. (2018). A systematic approach to the unconscious patient. *Clinical Medicine*.

[32] Knaus W. A., Draper E. A., Wagner D. P., Zimmerman J. E. (1985). Apache II: a severity of disease classification system. *Critical Care Medicine*.

[33] Bouch D. C., Thompson J. P. (2008). Severity scoring systems in the critically ill. *Continuing Education in Anaesthesia, Critical Care & Pain*.

[34] Tian Y., Yao Y., Zhou J., Diao X., Chen H., Cai K. (2022). Dynamic Apache II score to predict the outcome of intensive care unit patients. *Front. Med*.

[35] Munyua B. K. (2018). Evaluating the Validity of Apache Ii as a Predictor of Icu Mortality for the Critically Ill Patients at Knh’scritical Care Units.

[36] van der Merwe E., Kapp J., Pazi S. (2020). The SAPS 3 score as a predictor of hospital mortality in a South African tertiary intensive care unit: a prospective cohort study. *PLoS One*.

[37] Kruisselbrink R., Kwizera A., Crowther M. (2016). Modified early warning score (MEWS) identifies critical illness among ward patients in a resource restricted setting in kampala, Uganda: a prospective observational study. *PLoS One*.

[38] Ho L. O., Li H., Shahidah N., Koh Z. X., Sultana P., Ong M. E. H. (2013). Poor performance of the modified early warning score for predicting mortality in critically ill patients presenting to an emergency department. *World Journal of Emergency Medicine*.

[39] Klinger A., Mueller A., Sutherland T. (2021). Predicting mortality in adults with suspected infection in a Rwandan hospital: an evaluation of the adapted MEWS, qSOFA and UVA scores. *BMJ Open*.

[40] Kim D. Y., Lee M. H., Lee S. Y., Yang B. R., Kim H. A. (2019). Survival rates following medical intensive care unit admission from 2003 to 2013: an observational study based on a representative population-based sample cohort of Korean patients. *Medicine (Baltimore)*.

[41] Gayat E., Cariou A., Deye N. (2018). Determinants of long-term outcome in ICU survivors: results from the FROG-ICU study. *Critical Care*.

